# Respiration Subservient to Nutrition

**Published:** 1853-07

**Authors:** 


*Respiration subservient to Nutrition.*—A Thesis presented to the Medical Faculty of Harvard University, March, 1850. By E. LEIGH, M. D., Townsend, Mass.

THE above is the title of a Thesis, which, as appears from the title page, was presented to the Medical Faculty of Harvard University, in 1850. The object of the writer in permitting this production of his to appear in print, more than three years after its presentation to the above named honorable Faculty, is not as apparent to us as it might be, perhaps, were we better acquainted with the circumstances and influences by which he has been thus recently made mindful of a long neglected duty; for his views upon the respiratory functions, though passably well expressed, are not new or materially different from those of all well informed Physiologists of the present day. H.

---

WE are gratified, as we are sure our readers will be, in being able to present in this number of the Journal an original communication from Dr. Marshall Hall. Dr. Hall is now making a tour through the United States, and during his recent visit in this city performed several experiments illustrating his doctrine of the spinal system. We shall publish an account of them in our next. J.

